# Differences in the cytogenetic underpinnings of AL amyloidosis among African Americans and Caucasian Americans

**DOI:** 10.1038/s41408-022-00697-3

**Published:** 2022-07-04

**Authors:** Andrew Staron, Luke Zheng, Gheorghe Doros, Vaishali Sanchorawala

**Affiliations:** 1 Amyloidosis Center, Boston University School of Medicine, Boston, MA USA; 2 Section of Hematology and Medical Oncology, Boston University School of Medicine and Boston Medical Center, Boston, MA USA; 3 Department of Biostatistics, Boston University School of Public Health, Boston, MA USA

**Keywords:** Diseases, Myeloma

Dear Editor,

We previously observed that African Americans (AAs) with systemic light chain (AL) amyloidosis have an earlier age at diagnosis and a 24% higher risk of mortality after age-adjustment, compared to Caucasian Americans (CAs) [1]. Although these differences seemed to be at least in part explained by barriers to accessing care and lower use of stem cell transplantation among minorities, biologic predilections may also play a role. In multiple myeloma, younger age at disease onset among AAs and clustering of cases within families suggested an underlying genetic predisposition according to ancestry [2]. Efforts to characterize biological determinants of racial disparities in multiple myeloma revealed differences in cytogenetic architecture, with translocations t(11;14), t(14;16), and t(14;20) occurring more frequently and deletion 13q14 less frequently among individuals of African ancestry [3–6]. In one study, t(11;14) was found to be associated with shorter survival in AAs—a marked contrast to its neutral risk in the general multiple myeloma population [6].

AL amyloidosis and multiple myeloma have overlapping cytogenetic features, albeit the underlying plasma cell clone in AL amyloidosis is biologically distinct and more akin to monoclonal gammopathy of undetermined significance [7–9]. Its hallmark cytogenetic aberration is t(11;14), occurring in nearly half of patients with AL amyloidosis [9, 10]. Importantly, the presence of particular chromosomal abnormalities has prognostic implications for survival and treatment responsiveness [10–15]. Whilst understanding of the plasma cell clone and its clinical relevance in AL amyloidosis is expanding, these effects have not been investigated across diverse patient populations. Herein, we conducted a study of the biologic underpinnings of racial disparities in AL amyloidosis.

Newly diagnosed patients who underwent interphase fluorescence in situ hybridization (FISH) testing of bone marrow specimens for cytogenetic aberrations between January 2013 and January 2022 were identified from the prospectively maintained database at the Boston University Amyloidosis Center. Data accrual from consented patients was approved by the Institutional Review Board in accordance with federal regulations and the Declaration of Helsinki (ClinicalTrials.gov Identifier: NCT00898235). Inclusion required a diagnosis of systemic AL amyloidosis alone. Those with multiple myeloma-associated or lymphoplasmacytic lymphoma-associated AL amyloidosis were excluded due to distinct patterns of genetic aberrations in the accompanying disorders. Organ involvement, cardiac biomarker staging and hematologic responses were determined based on standard consensus criteria. Comparisons of clinicopathologic features between patient-identified racial groups (AAs vs. CAs) were performed by chi-square and one-way ANOVA tests, as appropriate. Overall survival (OS) was measured from the time of diagnosis to all-cause death or last follow-up (censored). The effect of t(11;14) on OS was assessed using multivariable Cox proportional hazards regressions with race, age, cardiac stage and use of high-dose melphalan and autologous stem cell transplantation (HDM/SCT) as covariates; and reported as hazard ratios (HR) with 95% confidence intervals (CI). Statistical tests were 2-sided with significance set at *P* < 0.050.

A total of 328 patients with systemic AL amyloidosis had cytogenetic testing, including 42 (13%) AAs and 286 (87%) CAs. Demographic and clinical characteristics upon baseline evaluation at the Amyloidosis Center were generally similar between racial groups (Table 1). Median age at diagnosis was 61 years for AAs, compared to 63 years for CAs (*P* = 0.077). The distribution and severity of organ involvement were non-significantly different. In the frontline setting, 13 (31%) AAs and 70 (24%) CAs received treatment with HDM/SCT; meanwhile, 22 (52%) AAs and 149 (52%) CAs received bortezomib-based regimens.

**Table 1:** Baseline characteristics and frequencies of chromosomal abnormalities in systemic AL amyloidosis according to self-identified racial group.

	African Americans (*n* = 42)	Caucasian Americans (*n* = 286)	Difference^a^	*P*
*Baseline characteristics*				
Median age, years (IQR)	61 (52–69)	63 (57–69)		0.077
Male, *n* (%)	24 (57)	170 (59)		0.777
λ amyloidogenic light chain, *n* (%)	34 (81)	232 (81)		0.980
Median dFLC, mg/L (IQR)	128 (48–262)	120 (62–289)		0.750
Median bone marrow plasma cells, % (IQR)	10 (10–18)	10 (5–15)		0.601
Heart involvement, *n* (%)	29 (69)	176 (62)		0.348
Median BNP, pg/mL (IQR)	194 (62–648)	219 (76–593)		0.758
BNP-based cardiac stage, *n* (%)				0.572
Stage I	13 (31)	76 (27)		
Stage II	19 (45)	119 (42)		
Stage III	3 (7)	43 (15)		
Stage IIIb	7 (17)	48 (17)		
Kidney involvement, *n* (%)	28 (67)	202 (71)		0.600
Median proteinuria, g/day (IQR)	3.2 (0.3–6.6)	2.9 (0.2–7.4)		0.382
*First-line treatment, n (%)*				0.621
Bortezomib-based regimen	22 (52)	149 (52)		
HDM/SCT	13 (31)	70 (24)		
Other treatment	4 (10)	38 (13)		
No treatment recorded^b^	3 (7)	29 (10)		
*Cytogenetic abnormalities, n (%)*				
Any cytogenetic aberrancy detected	36 (86)	219 (77)	9%	0.184
IgH translocations:				
t(11;14)	26 (62)	131 (46)	16%	0.051
t(4;14)	0 (0)	7 (2)	−2%	0.305
t(14;16)	2 (5)	2 (<1)	4%	0.025
t(14;20)	1 (2)	3 (1)	1%	0.463
Unknown partner	4 (10)	18 (6)	4%	0.435
Any IgH translocation	32 (76)	157 (55)	21%	0.009
Deletions:				
13q14	14 (33)	90 (32)	1%	0.808
17p13	1 (2)	5 (2)	0%	0.775
1p	1 (2)	1 (<1)	1%	0.114
Gains:				
1q21^c^	4/35 (11)	48/224 (21)	−10%	0.170
Any trisomy	13 (31)	97 (34)	−3%	0.704
Hyperdiploidy^d^	3 (7)	37 (13)	−6%	0.284

*IQR* interquartile range, *dFLC* difference in the involved and uninvolved light chains, *BNP* B-type natriuretic peptide, *HDM/SCT* high-dose melphalan and autologous stem cell transplantation, *IgH* immunoglobulin heavy chain, *CI* confidence interval, *NR* not reached.

^a^Difference signifies the frequency of the respective chromosomal abnormality among CAs subtracted from that among AAs.

^b^No treatments recorded due to early death (*n* = 10); loss to follow-up (*n* = 8); lack of vital organ involvement (*n* = 8); patient choice (*n* = 4) or therapy not yet initiated (*n* = 2).

^c^The probe for 1q21 gain was unavailable in 69 cases.

^d^Hyperdiploidy was defined as trisomies of at least two of the chromosomes 5, 9 or 15 per Wuilleme et al. [16].

At least one primary cytogenetic alteration was detected in 255 (78%) patients. In parallel with prior investigations [9, 10], the most prevalent alterations were t(11;14), deletion 13q14 and any trisomy (Table 1). Cytogenetics that are traditionally high-risk in multiple myeloma such as t(4;14), t(14;16) and deletion 17p13 were infrequent in our cohort. The aberrancy with greatest percentage difference between racial groups was t(11;14), present in 26 (62%) AAs vs. 131 (46%) CAs; yet, the threshold for statistical significance was not met (*P* = 0.051). Any immunoglobulin heavy chain (IgH) translocation was observed in 32 (76%) AAs and 157 (55%) CAs, representing a 21% higher frequency among AAs (*P* = 0.009). There was a suggestion of greater prevalence of 1q21 gain among CAs (21% vs. 11%; *P* = 0.170). Other aberrancies such as deletion 13q14 and chromosomal trisomies were similar between groups.

Heterogeneity in cytogenetic signatures could underly some of the survival disparities in AL amyloidosis. Thus, we analyzed the effect of t(11;14) status on OS between racial groups. At data cutoff date (January 2022), 114 (35%) patients were deceased. After adjusting for age, cardiac stage and use of HDM/SCT in a Cox model (Table 2), the hazard of death was estimated to be higher for AAs vs. CAs, although statistically non-significant (HR, 1.51; 95% CI, 0.85–2.69; *P* = 0.162). This disparity was not diminished after further adjusting for t(11;14) status (HR, 1.62; 95% CI, 0.90–2.92; *P* = 0.107), suggesting that this cytogenetic feature did not account for the survival disparity observed in our cohort. Presence of t(11;14) correlated with lower risk of mortality among CAs (HR, 0.68; 95% CI, 0.45–1.03; *P* = 0.067), whereas it seemed to have a more limited effect among AAs (HR, 0.88; 95% CI 0.31–1.53; *P* = 0.817). Moreover, AAs with t(11;14) were at an increased risk of early mortality, defined as death within 6 months of diagnosis, with a rate of 21% as compared to 13% for AAs without this translocation. A test for interaction (i.e., effect measure modification) between race and t(11;14) status using a Cox model was non-significant (*P* = 0.919).

**Table 2:** Cox proportional hazard regression analysis of all-cause mortality for African American (AA) race, with adjustment for clinical factors and t(11;14) status.

	Unadjusted		Model 1		Model 2	
	HR (95% CI)	*P*	HR (95% CI)	*P*	HR (95% CI)	*P*
**AA race**^a^	**1.07 (0.63–1.82)**	**0.808**	**1.51 (0.85–2.69)**	**0.162**	**1.62 (0.90–2.92)**	**0.107**
Age			1.04 (1.02–1.06)	0.001	1.04 (1.02–1.07)	<0.001
BNP-based cardiac stage ≥III			2.42 (1.60–3.65)	<0.001	2.45 (1.62–3.69)	<0.001
HDM/SCT-treated			0.42 (0.24–0.75)	0.003	0.44 (0.25–0.79)	0.006
Presence of t(11;14)					0.72 (0.48–1.07)	0.107

Bold values denote HR for AA race.

*OS* overall survival, *HR* hazard ratio, *CI* confidence interval.

^a^The reference group is Caucasian American race. Model 1 adjusted for age, B-type natriuretic peptide (BNP)-based cardiac stage, and use of high-dose melphalan and stem cell transplantation (HDM/SCT). Model 2 added t(11;14) status.

In an exploratory analysis of hematologic responses (i.e., very good partial response [VGPR] or better) to frontline treatments between racial groups, there was suggestion of higher response to HDM/SCT among AAs (11/13 [85%] vs. 40/62 [65%]; *P* = 0.158) and higher response to bortezomib-based regimens among CAs (83/126 [66%] vs. 8/17 [47%]; *P* = 0.130). While this analysis was limited in detecting differences due to the low number of AA patients included, the seeming divergence in treatment success between racial populations may in part be explained by differences in cytogenetic underpinnings. Prior investigations showed that patients with AL amyloidosis who harbor the t(11;14) translocation have inferior responsiveness to bortezomib-based regimens and superior responsiveness to HDM/SCT [10, 12–14]. These effects may be more pronounced among AAs, who have a higher frequency of IgH translocations. Moreover, this observation draws attention to the need to recruit members from underrepresented groups into clinical trials, as they may have different treatment success rates due to distinct cytogenetic underpinnings. Results from trials composed predominantly of CA participants may not accurately reflect treatment outcomes in patients of African ancestry.

In summary, our study indicates that, while the cytogenetic underpinnings of AL amyloidosis are generally similar across racial groups, IgH translocations are potentially more prevalent among AAs with AL amyloidosis—similar to prior observations in multiple myeloma [3–6]. Although this correlation may be mediated by ancestry, it is important to acknowledge that patient-identified racial categories are social constructs, which rely on a combination of skin color, geographic location, culture and religion. We conclude that these biological factors do not account for racial disparities in health outcomes—other influences such as socioeconomic factors [1] are likely to be more important contributors to racial disparities in AL amyloidosis.

## Data Availability

The datasets, including individual participants’ data supporting the results reported in this article will be available 3 months from initial request to researchers who provide a methodologically sound proposal. The data will be provided after its de-identification, in compliance with applicable privacy laws, data protection, and requirements for consent and anonymization.
